# Sex differences in the lateralization of sustained electrophysiological response to 40 Hz clicks in typically developing preschoolers

**DOI:** 10.1038/s41598-025-08266-x

**Published:** 2025-08-20

**Authors:** Victoria Manasevich, Anastasia Neklyudova, Olga Sysoeva

**Affiliations:** 1 Center for Cognitive Sciences, Sirius University of Science and Technology, Sirius, Russia; 2 Institute of Higher Nervous Activity and Neurophysiology, RAS, Moscow, Russia

**Keywords:** Language, Neurophysiology, Cortex

## Abstract

Two EEG-responses to periodical auditory 40-Hz stimulation – auditory steady-state response (ASSR) and sustained wave (SW) – are important neurophysiological correlates of temporal and spectral aspects of auditory perception, respectively. However, little is known about the sex difference of their development in children of 3–6 years, the period of extensive language acquisition. We registered a 32-channel electroencephalogram (EEG) while 500-ms 40-Hz click trains (*n* = 150) were presented to 57 typically developing children (27 females). We also assessed total language skill, expressive and receptive speech abilities, the perception of speech in noise and pseudowords repetition. 40-Hz ASSR was not very clearly expressed in our sample but showed developmental increase for both boys and girls. Topography of SW showed significant right-hemisphere predominance in boys, while girls had more symmetric response. On a subsample of boys (*n* = 14) we also found that the laterality index of SW correlated with speech in noise repetition score: the greater rightwarded the laterality was, the worse boys repeated words in noise. Our results are also consistent with the findings of greater lateralization of some components of EEG-activity in men.

**Supplementary Information:**

The online version contains supplementary material available at 10.1038/s41598-025-08266-x.

## Introduction

Differences between men and women span physiological, emotional, and cognitive domains^1^. Meta-analyses have shown that men outperformed women in 3D mental rotation, while women excelled in verbal fluency^2,3^. Research indicates that girls tend to acquire verbal skills more rapidly, particularly showing an advantage in phonemic fluency^4^, implying that women’s auditory processing of speech sounds may function differently than that of men.

A significant portion of the known sex differences indeed relates to auditory perception. These differences include low-level processes, such as longer Auditory Brainstem Response (ABR) latencies^5^ and lower temporal order thresholds in men compared to women^6^. There are also differences in more complex aspects of auditory perception, namely better binaural beat detection in men^7^ and better recognition of familiar melodies in women^8^.

While most studies were conducted in adults, several studies have identified sex differences in auditory processing in children and adolescents. It is of particular importance as sex differences can vary across different age groups. A longitudinal study, for instance, found that females scored significantly higher than males on both verbal and non-verbal abilities at ages 2, 3, and 4. However, at ages 10 and 12, males scored significantly higher than females on verbal ability^9^. Neurophysiological studies have shown that early components of the auditory response to tones were observed to increase in girls between the age ranges of 7–8 and 9–10 years, but no similar changes were noted in boys^10^. In adolescents (13–18 y.o.) early components were increased in males compared to females^11^. The literature on sex differences in early development however remains limited, and this study aims to help fill that gap.

Understanding sex differences in auditory perception during development is also crucial due to the disproportionate prevalence of neurodevelopmental disorders between sexes. Men are more frequently diagnosed with language impairments and also voice and speech-sound disorders^12–14^. Another neurodevelopmental disorder, which is more likely to occur in men is autism spectrum disorder (ASD)^15^. Given that auditory perception is frequently affected in ASD^16^, it is crucial to assess whether the developmental trajectories of brain responses to auditory stimuli differ between boys and girls.

Speech perception development in 3–6-year-old children represents a critical window in human language acquisition that warrants focused research attention. This developmental period is characterized by remarkable advances in children’s ability to perceive, process, and interpret spoken language, making it particularly significant for understanding the foundations of communication skills. By age 3, most children have progressed beyond babbling and single-word utterances to more complex linguistic structures, entering a phase where they can process multisyllabic words and increasingly sophisticated speech sounds^17^. During the period between 3 and 6 years, children develop crucial capabilities such as enhanced phoneme discrimination, even in challenging acoustic environments, improved word recognition and developing ability to perceive speech in noisy conditions^18^. Research highlights that children in this age group undergo significant development in audiovisual integration for speech perception. They increasingly utilize visual information, such as lip movements and facial expressions, to support language comprehension, particularly in challenging listening environments^19^. Older preschoolers (ages 5–6) show higher scores in receptive and expressive language with greater vocabulary diversity compared to younger children, indicating steady progression in both vocabulary breadth and depth during these years^20^. Summing up, studying speech perception in 3–6-year-old children provides a crucial window into how humans develop the fundamental ability to process and understand language – a cornerstone of human cognition and social interaction.

Brain responses to rhythmic 40-Hz stimulation may provide valuable insights into auditory perception in children. Such stimulation elicits two types of brain responses. The first one – auditory steady-state response (**ASSR) —** is an electrophysiological response, which follows the frequency of the stimulation^21^. This response is associated with fine temporal analysis of the signal^22,23^, which can be crucial for the detection of subtle differences in the speech signal. It has also been associated with speech in noise perception in young and old adults^24^. ASSR is often described in the literature as an EEG marker of schizophrenia^25^ and in some studies as a possible EEG marker of ASD^26,27^. However, an increasing number of studies comparing ASSR in typically developing children and children with ASD has not obtained significant results, at least not until the age of 14 years^28,29^.

The second brain response to rhythmic stimulation **– sustained wave** (SW) — occurs when rhythmic stimulus begins to be heard as continuous sounds with pitch and is described in the literature as a component associated with spectral auditory processing^30–32^. It may reflect the activity of the rate-coding neurons in the pitch-processing center^33^. SW is impaired in both autism spectrum disorder (ASD) and Rett Syndrome^34,35^.

Although these two auditory responses have been linked to ASD, a condition more prevalent in males, there is limited research on sex differences in these responses, particularly in early childhood. To address the lack of research, this study investigated the characteristics of ASSR and SW in boys and girls aged 3 to 6 years. Furthermore, we assessed various aspects of speech, including total language skill, expressive and receptive speech abilities, perception of speech in noise, pseudowords repetition. EEG responses described in this study are often associated both with the phonological side of speech perception^36^ and higher-level language impairments in neurodevelopmental disorders^35,37^.

## Results

### Questionnaire

Parents or legal guardians filled online questionnaires that estimated demographic characteristics of their children. According to the questionnaires, groups of boys and girls did not differ significantly by age (t(55)=−0.65, *p* = 0.518), height at birth (t(52)=−1.27, *p* = 0.209), weight at birth (t(52)=−0.679, *p* = 0.500), gestational age at birth (t(51)=−0.529, *p* = 0.599), frequency of left- or right-hand use (t(52) = 1.28, *p* = 0.207), economic status (income per family member) (χ2(7) = 6.95, *p* = 0.434) and mother’s education level (χ2(6) = 6.19, *p* = 0.402).

### Speech

We estimated speech development using Preschool Language Scales, 5th edition (PLS-5), a method that was designed to assess speech and the prerequisites for speech development for children from 0 to 7 years 11 months and included two scales to assess receptive and expressive speech^38^. There were no significant differences estimated with Student’s t-test between males and females for any of the scales. We also estimated speech in noise perception by presenting separate words in white noise of constant SNR and asking participants to repeat what they heard (see Methods for details). To estimate performance, we then calculated Levenshtein distance (LD) between the original word and answers of the children. No significant differences between LD in girls and boys were found. We also conducted a pseudoword repetition test where participants listened to pseudowords pre-recorded by a female Russian speaker and had to repeat what they heard. The number of correctly repeated words did not significantly differ between boys and girls. All behavioral results are presented in Table 1.

**Table 1:** Means, standard deviations (SDs) and t-test results for behavioral speech methods.

	Mean males (SD)	Mean females (SD)	t(df), *p*-value
General level of language competence (PLS-5 total language)	94.43 (17.05)	98.04 (19.54)	t(54) = 0.737, *p* = 0.464
PLS-5 receptive scale, raw score	54.57 (6.45)	54.58 (6.25)	t(54) = 0.006, *p* = 0.995
PLS-5 receptive scale, standard score	97.07 (15.20)	101.31 (15.22)	t(54) = 1.04, *p* = 0.303
PLS-5 expressive scale, raw score	50.93 (11.53)	49.35 (13.862)	t(54)=−0.468, *p* = 0.642
PLS-5 expressive scale, standard score	92.67 (19.3)	94.73 (23.28)	t(54) = 0.363, *p* = 0.718
Pseudowords repetition	15.41 (4.66)	15.00 (6.61)	t(48)=−0.255, *p* = 0.8
Levenshtein distance (Speech in noise)	1.63 (0.61)	1.65 (0.88)	t(28) = 0.06, *p* = 0.953

### EEG

#### Auditory steady-state response (ASSR)

**Fig. 1:**
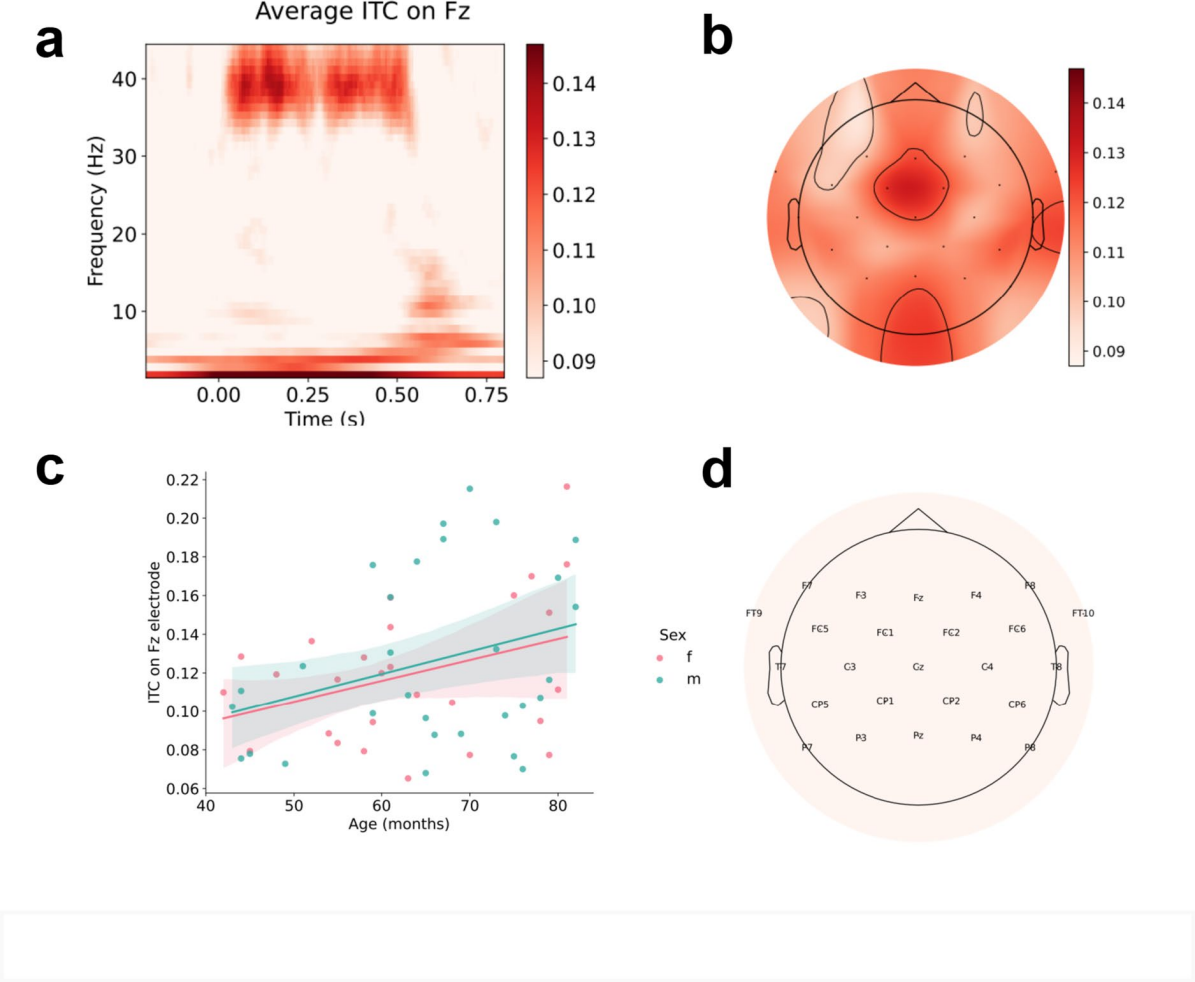
Grand-average inter-trial coherence (ITC) on Fz **(A)**, ITC topography within 39–41 Hz within 0–500 ms post-stimulus time window **(B)** and scatterplot of these ITC values on Fz in relation to age **(C)** with red and blue dots representing girls and boys, respectively. **(D)** reflects the electrode location.

We recorded 32-channel EEG while presenting 40-Hz click trains (*n* = 150) to our participants via headphones 65 dB SPL with inter-stimulus intervals varied between 500 and 800 ms. Inter-trial coherence (ITC) in the 39–41 Hz range, within 0–500 ms post-stimulus, and Fz channel was used in further analysis based on grand-average response (Fig. 1) and previous literature^34,35,61–63,66–68^, see “Methods”). Since the study included data from children aged 3 to 6 years, where the response might be negligible, it was important to examine if ASSR was different from zero. Results of this analysis showed a significant difference from zero (t(56) = 22.63, *p* < 0.001). To estimate if there are any dependencies on age we performed ANCOVA model with Sex as a between-group factor and Age (in months) as a continuous covariate. This analysis showed a significant effect for Age as a covariate (F(1,54) = 6.601, *p* = 0.013, pes = 0.109). To confirm the direction of the effect the correlation analysis was performed. The analysis showed that ASSR increases along with an increase in age (Fig. 1). Effect of Sex was not significant (F(1,54) = 0.141, *p* = 0.709, pes = 0.003). In our sample, ITC did not show significant correlations for speech parameters, including the Levenshtein distance (*r* = 0.57, *p* = 0.764, *n* = 30).

#### Sustained wave (SW)

**Fig. 2:**
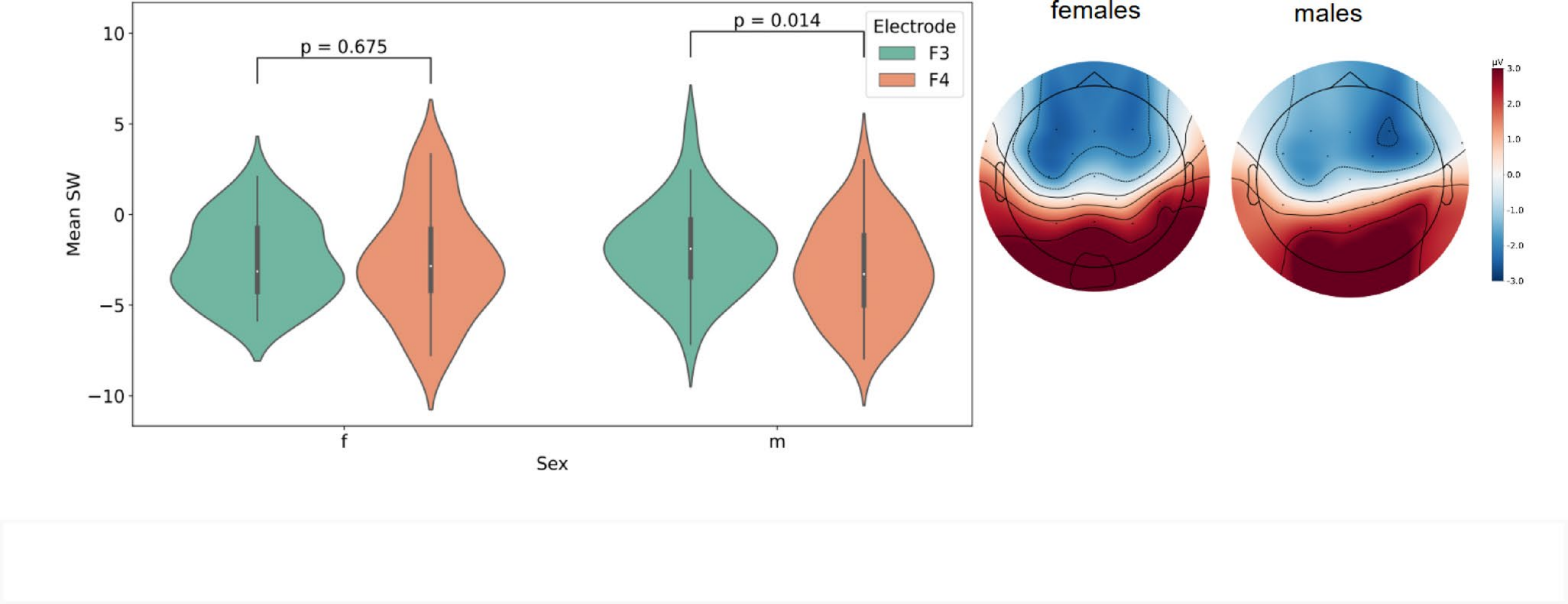
Amplitude and topography of sustained wave (SW) on F3 and F4 in females vs. males.

**Fig. 3:**
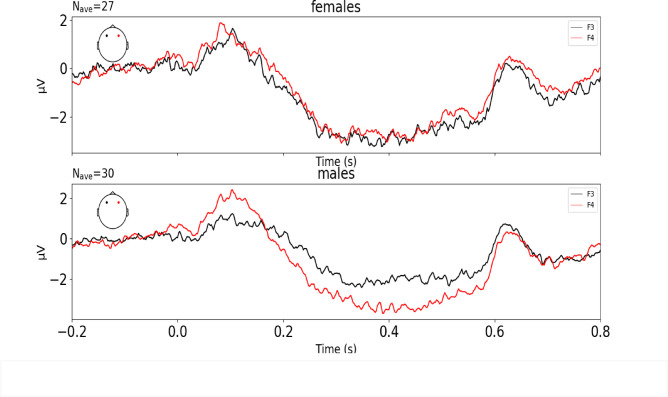
Grand-average ERP with sustained wave (SW) on F3 and F4 (black and red colors, respectively) in females (upper panel) and males (lower panel).

For the sustained wave, the mean ERP amplitude within the 250–550 ms post-stimulus range was used in further analysis to exclude the impact of early ERP components. Based on the topography of the sustained negative wave (Fig. 2) as well as previous studies^34–36,38,39^, see “Methods”), the electrodes F3 and F4 were used in statistical analysis. To estimate any statistical differences between sexes we used ANCOVA with Electrode as a within-group factor, Sex as a between-group factor and Age (in months) as a continuous covariate. A stable sustained wave was clearly expressed in the participants (Fig. 3). ANCOVA showed significant effect for Electrode*Sex interaction (F(1,54) = 4.231, *p* = 0.045, pes = 0.073). Other factors and interactions between factors were not significant Electrode*Age: F(1,54) = 0.119, *p* = 0.732 pes = 0.002; Age: F(1,54) = 0.711, *p* = 0.403, pes = 0.013; Sex: (F(1,54) = 0.047, *p* = 0.830, pes = 0.001, and Electrode: F(1,54) = 0.351, *p* = 0.556, pes = 0.006.

To elucidate the interaction between Electrode and Sex, for means comparison, we performed t-test for both groups (boys and girls) to compare SW values in each electrode. In males, we observed more pronounced SW in F4 than in F3 (t(29) = 2.609, *p* = 0.014) and, on the contrary, the difference between electrodes in females was not significant (t(26)=−0.424, *p* = 0.675). At the same time, for each electrode separately, t-test did not show significant results in males vs. females (for F3: t(55)=−1.3, *p* = 0.199, for F4: t(55) = 0.833, *p* = 0.409).

### Lateralization and speech

**Fig. 4:**
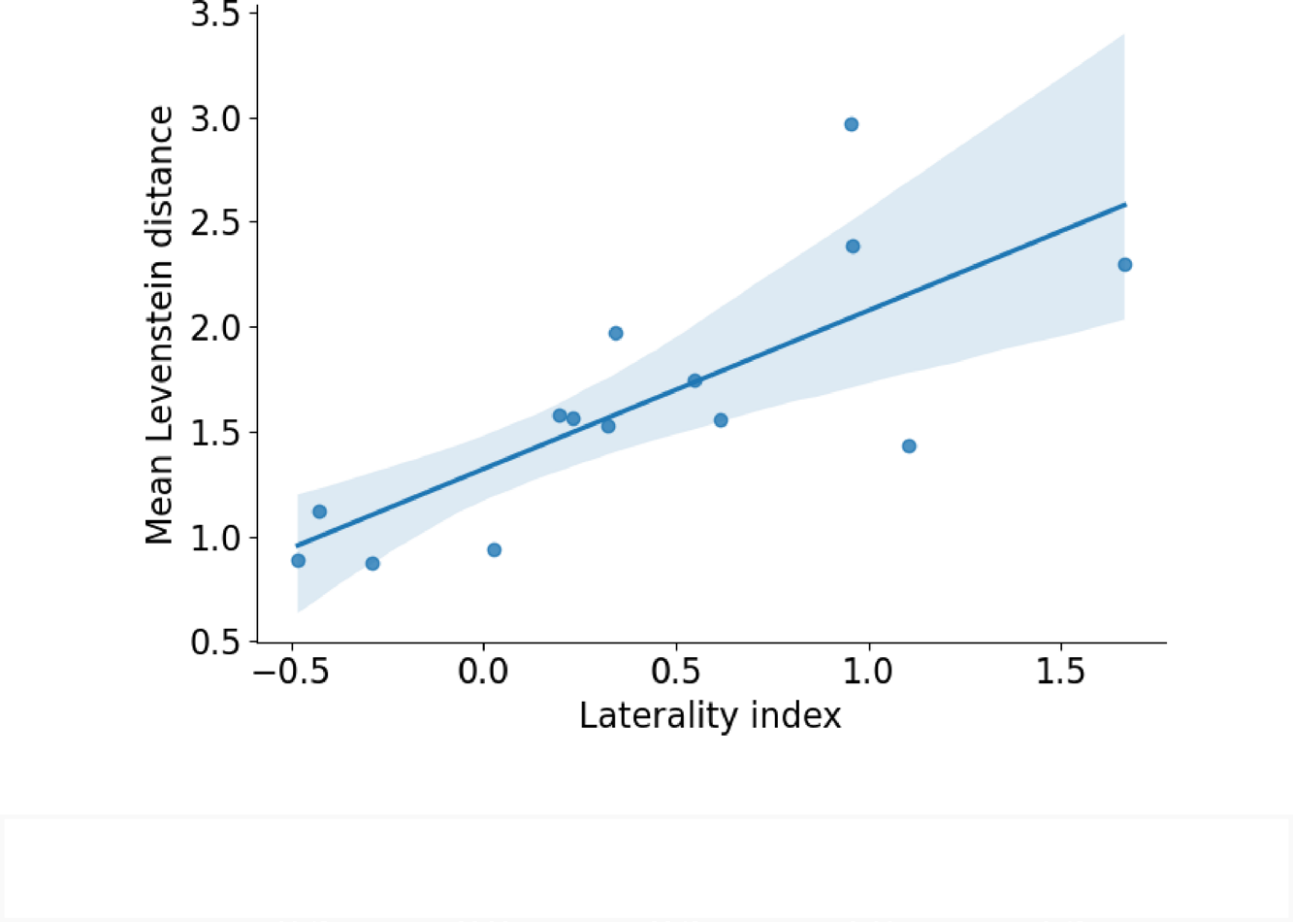
Relationship between results of speech in noise test and lateralization of sustained wave in males. Results of the test are presented as a mean Levenshtein distance within a participant. Levenshtein distance measures the number of errors between heard and repeated words.

We also calculated the laterality index (LI) for the sustained wave using the formula (SW amplitude F4 - SW amplitude F3)/(SW amplitude F3 + SW amplitude F4). We checked if LI depends on speech skills in children. We did not find any correlation for any scales of the PLS-5 test and also for the pseudoword repetition test. However, we found that in males (*n* = 14) the laterality index significantly positively correlated with results of speech-in-noise test, e.i. Levenshtein distance: *r* = 0.698, *p* = 0.012 (Fig. 4). The greater was the predominance of the right hemisphere over the left in boys, the poorer was their ability to recognize speech in noise.

## Discussion

In this study, we observed different topographies of the sustained wave (SW) in boys and girls aged 3–6 years. Boys showed a right-hemisphere predominance, while in girls this component was symmetrical. At the same time, rightward dominance in boys was associated with poor performance in the speech in noise task. Regarding the auditory steady-state response (ASSR, in our study operationalized as ITC), our study did not find any sex difference in this component, however confirmed previously found increase of ASSR with age. Here, in the Discussion section, we will review our results in light of findings in developmental neuroscience and their potential meaning for the research of neurodevelopmental disorders.

Our study confirmed the fact that ASSR increases with age. While we detected an ASSR response in our study, it was of small size, which is consistent with previous studies that showed that an ASSR response fully develops in children closer to 12 years^28,29,39,40^. The absence of sex differences in the features of this component at a frequency of 40 Hz has been previously shown in adults^25,41^. We expanded upon this finding by studying children aged 3–6 years. Although this component is still developing and small at this age range, our results suggest that ASSR develops at a similar rate in both boys and girls. Probably also due to rather weak ASSR in our studied group, we did not confirm the previous results of its relation to speech-in-noise perception, previously shown for young and old adults^24^.

This study was the first to describe SW in children as young as three years old. Studying this age group (3–6 y.o.) is crucial for several reasons. This period is marked not only by critical milestones in speech development but also by the emergence of symptoms associated with developmental disorders, such as autism spectrum disorder^42^. Contrary to ASSR, whose amplitude is still of a small size at this age and thus has limited utility as a diagnostic tool, a clear presence of SW is important for promoting this component as a potential early predictor of neurodevelopmental abnormalities. The pronounced SW suggests that spectral processing could be more important at this developmental period than the processing of fine temporal changes that are associated with ASSR^23,43^. Stroganova et al. (2020) have shown that the amplitude of a sustained wave in a similar paradigm was reduced in boys with autism spectrum disorder (ASD) of 7–12 years of age^34^. The sample of that study consisted of boys only, therefore it was not possible to study the sex differences in this component. However, in boys SW had a rightward dominance, identical to our findings. Building upon this finding, we were the first to compare the SW between typically-developing boys and girls and to show its greater rightward dominance in boys as early as 3–6 years of age. This is also consistent and extends the previous findings of greater lateralization of some ERP components in men than in women in auditory and other modalities^44,45^.

We also found a connection between the level of lateralization of SW and the level of speech in noise repetition in boys: the lower the rightward predominance index was, the more accurate children repeated words in noise. No sex difference, however, was found at behavioural level for this and other speech-related tests (general speech skills, both receptive and expressive, pseudowords repetition), pointing that boys and girls might differ in strategies of language learning and not its efficiency. As for the laterality index, our data expand previous results^44,45^, showing that men have a more asymmetric functional organization of the brain, including speech processing^10,11^. In this regard, we assumed that early speech processing can be reflected in the sustained wave, the components that similarly to ASSR is elicited by periodic sound stimulation. These processes may be differentially represented in the hemispheres of boys and girls and could be associated with their performance in speech-in-noise perception tasks.

In previous studies, speech perception in noise was associated with the ASSR in adults^24^. While we did not confirm this for 3–6 years old children, our results also emphasize the role of SW, another component elicited by the same type of stimulation as ASSR, in speech perception development in this age group. SW reflects the ability of the brain to integrate rhythmic stimuli into a continuous unity and enable detection of differences between rhythmic structure^46,47^. This is an important feature to detect changes in prosody which is crucial for speech perception development^48,49^. While we did not observe difference in behavioural measures of speech-related abilities in our sample, recent meta-analysis report that verbal skills, such as verbal fluency and verbal-episodic memory, that was not examined in our study, develop more rapidly in girls compared to boys^4^. Our neurophysiological findings may provide some explanation for this phenomenon: boys who were better at speech perception in noise had more symmetrical, namely, “girl-like” SW topography in response to 40-Hz clicks trains. Future studies need to investigate the age at which this tendency emerges and whether it disappears after the critical period for speech development. Moreover, it would be interesting to examine if SW is involved in speech development in girls at younger ages.

It is known that the prevalence of ASD in boys and girls differ, with an average of 4 boys for every girl with ASD^50^. Furthermore, girls and boys with higher levels of autistic traits compared to their age- and sex-matched peers exhibit distinct phenotypes in expressive language development. Specifically, boys experience a temporary decline in expressive language around the age of two, followed by a slight improvement thereafter^51^. One of the crucial neurophysiological mechanisms of speech development in this period (after 2 y.o.) might be the one we described in this study: the development of the pitch processing mechanism. Rightward-lateralization of SW related to pitch processing, which appears to characterize typically-developing boys, may aid in identifying sex-related characteristics of phenotypes and inform future research on ASD biomarkers. In particular, Herringshaw and colleagues^52^ showed that people with ASD have what authors call “right hemispheric dominance” in speech processing within different speech domains: semantic processing, sentence comprehension, processing figurative language, and speech production. Altered functional lateralization of the language network in ASD may have different structure and different features depending on sex of an individual and, thus, demand different strategies during its compensation. We can speculate that the association between the laterality index of SW and speech-in-noise perception observed only in boys might indicate the susceptibility of pitch-related speech processing to impairment in this group, which is likely seen in ASD. This might subsequently help to shed light to cortical bases of language dysfunction, including those in neurodevelopmental disorders such ASD, which show different prevalence depending on sex.

### Limitations

Our study involved only children aged 3 to 6 years, in which the ASSR is not very clearly expressed. Therefore, all conclusions related to ASSR should be taken with caution. Also, a correlation between the index of laterality of the SW and the Levenshtein distance in boys was found in a subsample of 14 boys, so, this finding is more of an exploratory nature and should be confirmed on a larger sample.

## Conclusions

Our study of children aged 3–6 years old confirmed a previously described developmental increase of 40-Hz ASSR, brain response associated with temporal resolution of the auditory cortex, and did not reveal any sex difference. On the contrary, SW was pronounced and rather stable within the studied age range, which adds to its advantage as a putative early predictor of neurodevelopmental disorders. Topography of SW showed significant right-hemisphere predominance in boys, while girls had symmetric response. On a subsample of children, we also found that the laterality index of SW in boys correlated with speech in noise repetition score: the greater the predominance of right over the left hemisphere was, the worse males repeated words in noise. This finding can be associated with both the phenomenon of “right hemispheric dominance” in speech processing in people with ASD, which is most prevalent in boys, and the fact that more “girl-like” lateralization of SW, can give advantage in some speech perception processes and as a consequence in the development of language and speech. However further research (preferably longitudinal) is needed to prove these assumptions.

## Methods

### Participants

The study included 57 (27 females) typically developing children, defined as those free from developmental disorders and diagnosed neurological or psychiatric conditions, with ages ranging from 42 to 82 months and a mean age of 64.35 months (SD = 11.96). These children were screened for ASD and showed no signs of neurodivergence based on the questionnaire. In this study we focused on effects to be clinically significant, so the sample size was determined to be suitable for the analysis of strong effects (effect size f ≥ 0.4). Thus, according to the power analysis conducted using the G*power software^53^, the sample size exceeds the minimally required sample to detect a strong effect in ANCOVA – 52 (with following parameters effect size f of 0.4, alpha error probability of 0.05 and 80% power, number of groups – 2, numerator df – 1 and number of covariates – 1). Some behavioral methods were completed only by a subset of the participants in the overall sample because of difficulties in task performance and technical issues: speech in noise paradigm was completed by 30 participants (16 females, mean (SD) = 65.36 months (10.43), and pseudowords repetition test was completed by 50 participants (23 females, mean (SD) = 64.52 months (12.03)).

### Data collection and ethics

This study was conducted on the federal territory of Sirius and was approved by the Sirius University Bioethics Committee (Bioethics Committee Opinion dated 13.07.2022) in accordance with the principles outlined in the Declaration of Helsinki. Prior to data collection, informed consent was obtained from the participants’ parents, and verbal assent was obtained from the children participants. This study strictly adhered to the approved protocols of EEG and behavioral data collection and management.

### Questionnaires

Parents completed an online demographic questionnaire which contained 63 questions concerning the child’s health status, handedness, economic status of the family and some general questions. Children with no neurological/psychiatric disorders according to information from this questionnaire were included in this study.

### Behavioral methods

*PLS-5.* PLS-5 was given to assess speech and the prerequisites for speech development for children from 0 to 7 years 11 months and included two scales to assess receptive and expressive speech^38^. The PLS-5 methodology (The fifth edition) has been translated and adapted into Russian by researchers from St. Petersburg State University^54,55^. The PLS-5 toolkit is a set of toys and a guide with pictures; the test is conducted in a semi-structured game form. The tasks of both scales are arranged in ascending order of difficulty and are divided by age^54^.

### Pseudowords repetition

For this study we used a modification of the pseudoword repetition test from ITOG – The standardized assessment of literacy skills in children, developed by the Laboratory for Interdisciplinary Research of Human Development in Saint Petersburg^56^. This study employed the computerized subtests of the ITOG: repetition of pseudowords^56^. Participants listened to pseudowords pre-recorded by a female Russian speaker and had to repeat what they heard. The recording was made on the computer’s built-in microphone. The number of correctly repeated words was then counted.

### Speech in noise

During this paradigm, the sequence of words was presented in the white noise. The words were spoken by a male Russian speaker. The total number of words in this paradigm was 40 Russian words varied from 1 to 4 syllables long. Signal to noise ratio was constant and remained at the level of 15 dB. Stimuli were presented via PsychoPy. Participants were asked to listen to words in white noise and repeat them. The words repeated by the participants were recorded with a built-in microphone and then transcribed by two independent experts for further analysis. In particular, we used the Latin alphabet for transcription and each Russian consonant had two-symbol correspondences: with index 1 for hard consonants and with index 2 for soft ones, the transcription rules we had developed earlier^57^. To measure the ability of speech in noise perception we used the Levenshtein distance — a metric of difference between two words on a symbol-by-symbol basis^58^. Within the speech in noise paradigm it can also be used to examine speech discrimination skills^59^. Levenshtein distance provides a sensitive measure of speech-recognition accuracy by calculating the minimum number of character edits needed to transform one word into another. Unlike pass/fail metrics, it awards partial credit when words are recognized with minor errors or position shifts. This nuanced approach captures subtle perceptual differences. This sensitivity makes Levenshtein distance particularly valuable for detecting gradients in speech recognition performance that binary measures might miss^60^. Successful performance of speech-in-noise repetition task requires correct perception of words in noisy background, as well as translation of this perception into a proper motor program. While we tried to exclude the motor dysfunction contribution to this process by accounting for individual characteristics of pronunciation and providing an opportunity to correct the answer, this test evidently captures not only pure speech-in-noise discrimination, but other speech-related processes as well. As speech repetition is a common approach to study language, we believe this test is ecologically valid to examine language skills in children.

### Stimuli and procedure of EEG experiment

Сlick trains (*n* = 150) at a frequency 40 Hz and duration of 500 ms were presented binaurally via headphones 65 dB SPL with inter-stimulus interval varied between 500 and 800 ms. The waveform of each click was a rectangular pulse of a 0.2-ms duration. The experimental sequence lasted approximately 3 min. During the presentation, participants were watching a muted video of their choice. Such an experimental procedure aimed to help the participants to sit still and feel calm and comfortable during the experiment as this is a standard procedure in this type of experiment.

### EEG-recording

EEG-data was acquired in a shielded chamber. A 32-channel EEG was recorded using a Brain Products actiCHamp system (Brain Products GmbH, Gilching, Germany) at a sampling rate of 50 kHz, in parallel with auditory stimulation. No online filters were applied (bandwidth range: DC to 10300 Hz). Reference electrode was set on the FCz position. Impedance was kept below 25 kΩ.

### EEG data preprocessing

Data were preprocessed with Python MNE-package v.1.3.0. Data were downsampled to 500 Hz. Visual check of noisy channels in raw data was made. Technical channels (such as microphone, photosensor) were deleted. Borderline channels (TP9, TP10, Fp1, Fp2, O1,O2) were deleted from the analysis during the preprocessing stage, because they showed a lot of artificial activity due to the high probability of muscle artifacts and the proximity to the headphones which might be touched by the participant. We did not discard any other channels within the preprocessing pipeline. Other channels with low signal to noise ratio were interpolated with default MNE-function. The number of interpolated channels for each participant did not exceed two. Then a mean reference was set for all the recordings. Recordings were epoched -200–800 ms around the event marker. Trials with an amplitude greater than 5 times the standard deviation (within each epoch for each channel) after this filtration were excluded from further analysis.

### EEG data processing

#### Auditory steady-state response (ASSR)

For this analysis, data were band-pass filtered from 0.1 to 80 Hz before epoching. As a measure for analysis we chose inter-trial coherence (ITC). ITC quantifies the consistency of neural responses across multiple trials. Because ITC measures phase locking of the neural oscillations to the auditory stimulus, this is particularly important, as it assesses how well the brain synchronizes its electrical activity with the frequency and phase of the auditory input, which is a key characteristic of auditory temporal processing^61^. ITC has been previously used to assess auditory processing capabilities in various clinical populations, such as individuals with psychiatric or neurological disorders and is a quite common measure for such studies^62,63^. The mean number of epochs taken into ITC analysis for each participant was 131.5 ± 9.34, range 108–147 epochs, in girls group 131.23 ± 8.86, range 108–144, in boys group 131.38 ± 9.66, range 112–147. We calculated inter-trial coherence for ASSR with the python MNE-function tfr_stockwell. Based on the topography of the ASSR in our data and previous literature^64–66^, the ITC value ​on electrode Fz was chosen for the analysis (Fig. 1). The Fz electrode provides a high signal-to-noise ratio for ASSR detection, especially for the 40 Hz response generated in auditory cortex and frontal regions^67,68^. Its location offers excellent sensitivity to cortical auditory responses, making it common in ITC ASSR studies^66–68^. High ITC values at Fz indicate stronger phase-locked responses, reflecting temporal processing in the auditory cortex, even in developing brains. The Fz electrode is valuable in clinical research and crucial for our investigations of periodical stimulation in children with ASD and developmental disorders, as 40 Hz ASSR recorded at Fz is sensitive to neuropsychiatric conditions like schizophrenia^68^. Inter-trial coherence in the 39–41 Hz range, within 0–500 ms post-stimulus, was used in further analysis as the 40 Hz-ASSR. In our paradigm with 500 ms click-train duration ASSR appears within tens of milliseconds after stimulation onset and lasts approximately 500 ms^34,35^.

### Sustained wave (SW)

For this analysis we did not apply any additional filter because according to existing literature the presence of low-pass filtration does not affect the resulting component in case of SW^35,36,38,39^. Sustained wave is a slow-wave oscillation by its nature and is not observed at high frequencies anyway. Moreover, since we used the average amplitude in the wide range as the analyzed parameter, the high frequencies did not have a significant effect on the final value. The absence (or at least very low) of high-pass filters is crucial for detection of SW as it is a low-frequency component. Epochs were averaged within the participant for this analysis to obtain event-related potential (ERP). The mean number of epochs taken into SW analysis for each subject was 123.04 ± 11.33, range 97–145 epochs, in girls group 123.04 ± 12.77, range 97–143, in boys group 122.28 ± 10.08, range 103–145. We did not analyze traditional early components of ERPs (N1, P1, etc.) as they are usually not pronounced under this type of stimulation. Based on the topography of the sustained negative wave, the electrodes F3 and F4 were selected as most representative. While most standard ERP protocols focus on central and midline electrodes, the inclusion of frontal electrodes F3 and F4 provides valuable additional information about lateralized neural activity, and a sustained wave is commonly detected in both hemispheres^34,35^. The mentioned studies considered FC3 and FC4 as electrodes of interest, however, in our 32-channel system the closest corresponding channels are F3 and F4. In addition, the response amplitudes in our data in these sites were the highest, as can also be seen on topography on Fig. 2. Also temporal electrodes (e.g., T7/T8) are prone to muscle artifacts (e.g., jaw/neck movements), whereas frontal electrodes (F3/F4) experience fewer such artifacts. For the sustained wave, the mean ERP amplitude within the 250–550 ms post-stimulus range was used in further analysis to exclude the impact of early ERP components and consistent with grand-average response (Fig. 2) and previous literature^34,35^. We also calculated the laterality index for the sustained wave using the formula (SW amplitude F4 - SW amplitude F3)/(SW amplitude F3 + SW amplitude F4).

### Statistical analysis

Statistical analysis was carried out using IBM SPSS 27. For ASSR analysis, we used a one-way ANCOVA model with Sex as a between-group factor and Age (in months) as a continuous covariate. Student’s one-tailed t-test was used to assess if the ASSR-response differed from zero. For SW, we used a two-way ANCOVA type 3 model with Electrode (F3 or F4) as a within-group factor, and Age (in months) as a continuous covariate and Sex as a between-group factor. We analyzed possible sex differences in parents’ reported parameters and behaviorally assessed speech parameters with Student’s two-tailed t-test for independent samples. Pearson correlation was used to test the relationship between behavioral and neurophysiological parameters.

## Electronic supplementary material

Below is the link to the electronic supplementary material.

Supplementary Material 1

## Data Availability

Data and materials supporting the results or analyses presented in this paper are available upon following link: https://doi.org/10.6084/m9.figshare.27225894.v1.
