# Supplementary figures and images for: Sex differences in the lateralization of sustained electrophysiological response to 40 Hz clicks in typically developing preschoolers
Source: Sci Rep. 2025 Aug 20;15:30548. doi: 10.1038/s41598-025-08266-x (PMC12368233; doi:10.1038/s41598-025-08266-x)

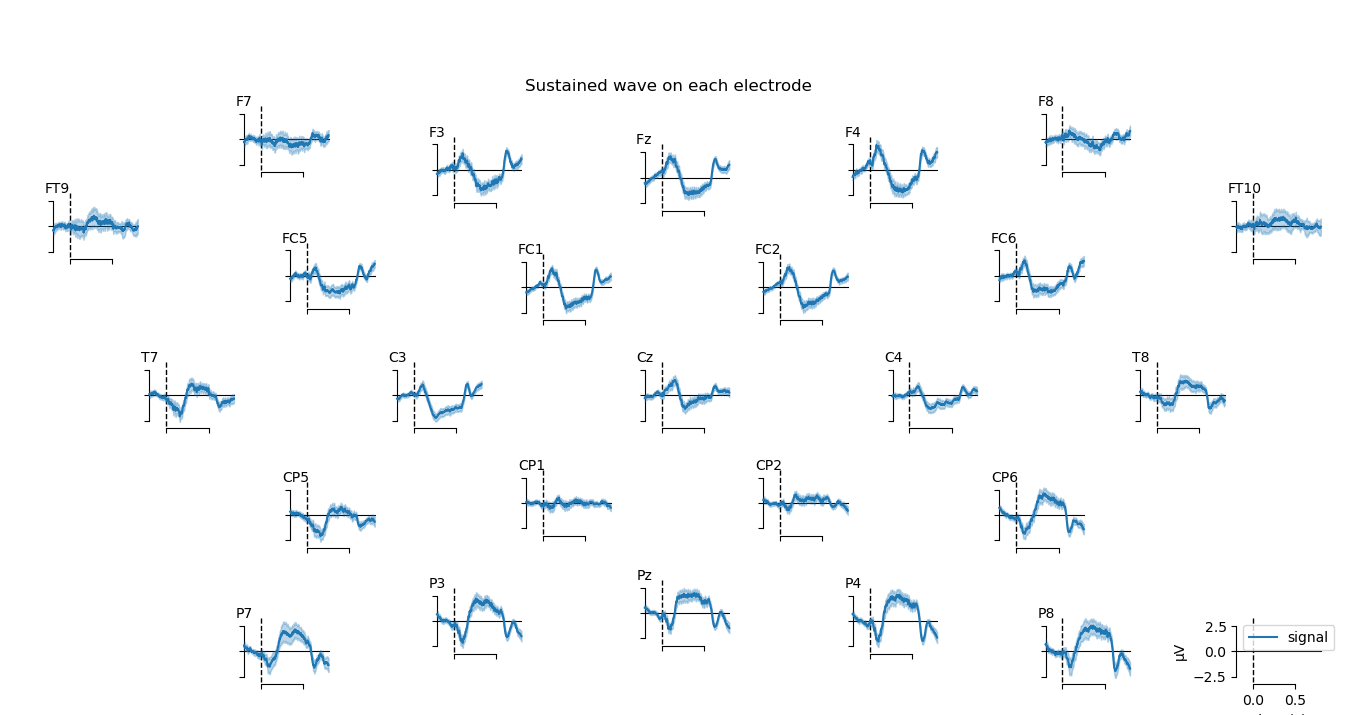


Fig. 1 Sustained wave on each site.

Supplement: Supplementary file 1 — Supplementary Material 1 [file 41598_2025_8266_MOESM1_ESM.docx]
